# *ERBB2*-Mutant Gastrointestinal Tumors Represent Heterogeneous Molecular Biology, Particularly in Microsatellite Instability, Tumor Mutation Burden, and Co-Mutated Genes: An In Silico Study

**DOI:** 10.3390/cimb45090468

**Published:** 2023-09-11

**Authors:** Shiro Uchida, Takashi Sugino

**Affiliations:** 1 Division of Diagnostic Pathology, Kikuna Memorial Hospital, 4-4-27, Kikuna, Kohoku-ku, Yokohama 222-0011, Japan; 2 Division of Pathology, Shizuoka Cancer Center, Shizuoka 411-8777, Japan; t.sugino@scchr.jp; 3 Department of Human Pathology, Juntendo University School of Medicine, Tokyo 113-8421, Japan

**Keywords:** esophageal adenocarcinoma, gastric cancer, colorectal cancer, *ERBB2* mutation, microsatellite instability, tumor mutation burden

## Abstract

During recent years, activating mutations in *ERBB2* have been reported in solid tumors of various organs, and clinical trials targeting *ERBB2*-mutant tumors have been conducted. However, no effective treatment has been established for gastrointestinal tumors targeting *ERBB2* mutations. *ERBB2*-mutant tumors have a higher tumor mutation burden (TMB) and microsatellite instability (MSI) than *ERBB2* non-mutant tumors, but not all *ERBB2*-mutant tumors are TMB- and MSI-high. Thus, a more detailed classification of *ERBB2*-mutant tumors based on the underlying molecular mechanisms is required. Herein, we classified *ERBB2* mutations into three groups—group 1: both *ERBB2* mutations and amplifications; group 2: *ERBB2* mutations annotated as putative driver mutations but without amplifications; group 3: *ERBB2* mutations annotated as non-driver mutations (passenger mutations or unknown significance) and those that were not amplified in gastrointestinal tumors. Esophageal adenocarcinoma, gastric cancer, and colorectal cancer presented significantly higher MSI and TMB in the *ERBB2*-mutant group than in the *ERBB2*-wild-type group. The proportions of TMB- and MSI-high tumors and frequency of co-mutated downstream genes differed among the groups. We identified TMB- and MSI-high groups; this classification is considered important for guiding the selection of drugs for *ERBB2*-mutant tumors with downstream genetic mutations.

## 1. Introduction

Recently, activating mutations in *ERBB2* have been reported in several solid cancers; they have been shown to play an oncogenic role similar to that of *ERBB2* amplifications [1,2,3]. In the gastrointestinal tract, studies have been conducted on *ERBB2*-mutant gastric cancer (GC) and colorectal cancer (CRC) [4,5]; however, none have comprehensively examined *ERBB2*-mutant esophageal adenocarcinoma (EAC). Trastuzumab has been found to be effective against HER2-positive EAC [6], GC [7], and CRC [8,9] in clinical practice. On the contrary, trastuzumab deruxtecan (DS-8201a) has been shown to be effective for ERBB2-mutant non-small-cell lung cancer [10]. However, there are no known effective treatments for ERBB2-mutant EAC, GC, and CRC. Therefore, in this study, we focused on the gastrointestinal tumors (EAC, GC, and CRC).

Candidate therapeutic agents targeting ERBB2 kinase activity include monoclonal antibodies, antibody–drug conjugates, and small-molecule tyrosine kinase inhibitors. Although there are several clinical trials of drugs targeting *ERBB2*-mutant tumors with accumulating data, research on targeting *ERBB2* mutations for cancer treatment has been slow owing to their low mutation frequency, insufficient understanding of their biological activity, and difficulty in detection [11]. In addition, drug susceptibility of cancer varies with *ERBB2* mutations [12], and identifying individual *ERBB2* mutations to develop effective targeted treatments is difficult. *ERBB2*-mutant tumors have a higher microsatellite instability (MSI) [13] and tumor mutation burden (TMB) than *ERBB2* non-mutant tumors [1,2,5,14], suggesting that immune checkpoint inhibitors may be useful against *ERBB2*-mutant tumors [2,5,13]. However, not all *ERBB2*-mutant tumors in previous studies were MSI- and TMB-high [1,2,5,13,14]. In addition, previous studies have suggested that drug sensitivity or resistance can be attributed to the changes in downstream or parallel oncogenic pathways rather than the mutations themselves [3]. Considering the drug sensitivity, microsatellite (MS) status, and TMB, *ERBB2*-mutant tumors are regarded as heterogeneous.

Here, we aimed to determine whether heterogeneous *ERBB2*-mutant tumors can be appropriately classified by biological features (clinicopathologic features, MS status, TMB, and co-mutated genes) to facilitate the selection of an appropriate treatment plan using bioinformatic methods. Furthermore, we discuss whether this classification would lead to effective therapies that consider the biological and co-mutated genetic characteristics of each group.

## 2. Materials and Methods

### 2.1. Data Collection of EAC, GC, and CRC Cases

Genomic and clinical data were collected from EAC, GC, and colorectal adenocarcinoma tumor cases using cBioPortal. Specifically, data from The Cancer Genome Atlas (TCGA) Pan-Cancer Atlas dataset (EAC) [15] and Memorial Sloan Kettering (MSK) dataset (esophagogastric cancer) [16] (*n* = 87 and 228, respectively) were obtained. Additionally, data from TCGA Pan-Cancer Atlas [15] and the OncoSG dataset [17] (*n* = 440 and 147, respectively) were collected for stomach adenocarcinoma, and data from TCGA Pan-Cancer Atlas [15] and the MSK dataset [18] (*n* = 594 and 471, respectively) were collected for colorectal adenocarcinoma. Next, samples from patients with EAC, stomach adenocarcinoma, and colorectal adenocarcinoma were divided into two groups each: those with *ERBB2*-mutant (*n* = 22, 38, and 39, respectively) and *ERBB2* non-mutant (*n* = 293, 549, and 1026, respectively) tumors. 

*ERBB2* mutation sites were classified as follows: receptor-L domain, furin-like cysteine-rich domain, growth factor receptor domain, transmembrane domain, juxtamembrane domain-A, juxtamembrane domain-B, kinase domain, and C-terminal region. The distribution of genetic mutation sites in each carcinoma group is summarized in Table S1. The pathological significance of each *ERBB2* mutation was assessed using the OncoKB database (Table S2) [19].

### 2.2. Molecular Subtype Classification

Molecular subtypes of EAC, GC, and CRC were determined on the basis of a previous study [20]. Using data from cBioPortal, five subtypes were identified: chromosomal instability (CIN), genome stability (GS), MSI, POLE, and Epstein–Barr virus (EBV). Specifically, EAC and CRC were classified into four types each—CIN, GS, MSI, and POLE—whereas GC was classified into five types—CIN, GS, MSI, POLE, and EBV.

### 2.3. MSI Analysis

For samples from TCGA Pan-Cancer Atlas, the MS status was assessed using an MSI sensor, a computational algorithm that analyzes sequencing reads at designated MS regions in tumor–normal tissue pairs, reporting the percentage of unstable loci as a cumulative score [21,22]. MSIsensor scores ≥10 are defined as MSI-H, scores ≥3 to 10 are defined as MSI-intermediate (MSI-I), and scores <3 are defined as microsatellite stability (MSS). In the esophagogastric cancer (MSK) and CRC (MSK) datasets, the MS status was divided into stable, indeterminate, and unstable. Therefore, in this study, MS status was categorized into MSS, MSI-I, and MSI-H. Information regarding the MS status was not available in the OncoSG dataset.

### 2.4. TMB Estimation

TMB is a measure of the total number of mut/Mb of the tumor tissue. It can also be interpreted as the mutation density in tumor genes, defined as the average number of mutations in the tumor genome, including the total number of coding sequence errors, base substitutions, insertions, or deletions. Information on TMB was obtained from all datasets. EAC and GC samples were classified as TMB-high if they had ≥10 mut/Mb and TMB-low if they had <10 mut/Mb [23]. In CRC, TMB ≥ 16 mut/Mb was classified as TMB-high, and TMB < 16 mut/Mb was classified as TMB-low [24,25].

### 2.5. Comparison of MS Status and TMB between ERBB2-Mutant and ERBB2-Wild-Type EAC, GC, and CRC

We conducted a comparative analysis of MSS, MSI-I, and MSI-H ratios between the *ERBB2*-mutant and *ERBB2*-non-mutant groups of EAC, GC, and CRC. Additionally, we compared the TMB of each cancer, as well as the TMB-low to TMB-high ratio between the *ERBB2*-mutant and *ERBB2*-non-mutant groups.

### 2.6. Comparison of Clinicopathological and Molecular Features among the Three Groups Each of EAC, GC, and CRC

EAC, GC, and CRC samples with *ERBB2* mutations were categorized into three groups each on the basis of OncoKB annotation and amplification status. Group 1 included samples with both *ERBB2* mutations and amplifications, group 2 comprised samples with *ERBB2* mutations annotated as putative driver mutations by OncoKB but without amplifications, and group 3 consisted of samples evaluated as non-driver mutations (passenger mutations or unknown significance) by OncoKB and those that were not amplified. We compared the clinicopathological and molecular characteristics of each of the three groups.

For EAC samples, information on age (mean), sex distribution, histological grade, and molecular subtype was sourced from cBioPortal for the respective cancer types. Cases for which stage information was not available were designated as “N/A” and excluded from percentage calculations. However, age information was not available for the esophagogastric cancer dataset (MSK).

Similarly, for GC samples, information on clinicopathological characteristics, age (mean), sex distribution, histological grade, and molecular subtype was obtained from cBioPortal. For certain variables, including age, sex, histological grade, and molecular subtype, information was not available in some cases, and these were designated as “N/A”. These cases were excluded from percentage calculations.

For CRC samples, information on clinicopathological characteristics, age (mean), sex distribution, histological grade, and molecular subtype was obtained from cBioPortal. Information regarding molecular subtypes was not available in the colorectal adenocarcinoma dataset (MSK). The MS status and TMB were categorized as described above for each cancer type.

### 2.7. Analysis of ERBB2 Amplifications and Mutations in Signaling Pathways in EAC, GC, and CRC Samples and Comparison of the Frequencies of Genetic Variants in Each Group

We used cBioPortal to investigate the following signaling pathway genetic mutations in EAC, GC, and CRC samples: RTK signaling (*ERBB2*, *ERBB3*, *ERBB4*, *EGFR*, and NTRK3), WNT/β-catenin signaling (*APC*, *CTNNB1*, and *RNF43*), TGF-β signaling (*SMAD4*), PIK/MTOR signaling (*PTEN*, *TSC1*, and *mTOR*), RAS/RAF/MAPK signaling (*KRAS*, *NRAS*, *NF1*, and *BRAF*), mismatch repair (*POLE*, *MSH2*, *PMS2*, and *MSH6*), homologous recombination (*BRCA2*, *ATM*, *PARP1*, and *RAD50*), epigenetic modifiers (*ADID1A*, *KMT2C*, and *KMT2D*), cell cycle (*TP53*), and *ERBB2* amplifications. The detected genetic alterations were visualized using Oncoprinter on cBioPortal. The number of mutations in each group was compared. All datasets only showed somatic mutations, and germline line mutations were not examined. Patients with Lynch syndrome may have been includincludeded.

### 2.8. Statistical Analysis

We tested the normality of continuous variables (TMB) using the Kolmogorov–Smirnov test in *ERBB2*-mutant EAC, GC, and CRC and *ERBB2*-wild-type EAC, GC, and CRC; no normality was confirmed in any of the cases. Therefore, the TMB in *ERBB2*-mutant and *ERBB2*-non-mutant samples was analyzed using the Mann–Whitney U test. The MS status and TMB in *ERBB2*-mutant and *ERBB2*-non-mutant samples were analyzed using chi-square. The clinicopathological and molecular features of groups 1, 2, and 3 of EAC, GC, and CRC were analyzed using Fisher’s exact test. The normality of the TMB values of the EAC, GC, and CRC groups was also verified using the Kolmogorov–Smirnov test. Considering the results of the test and the sample size, the TMB of groups 1, 2, and 3 of EAC, GC, and CRC was analyzed using Kruskal–Wallis test. Steel–Dwass post-test correction was used to reduce the likelihood of false positives. Statistical significance was set at *p* < 0.05. All statistical analyses were performed using EZR software (version 1.55) [26].

## 3. Results

### 3.1. Comparison of MS Status and TMB between ERBB2-Mutant and ERBB2-Wild-Type EAC, GC, and CRC

**Figure 1:**
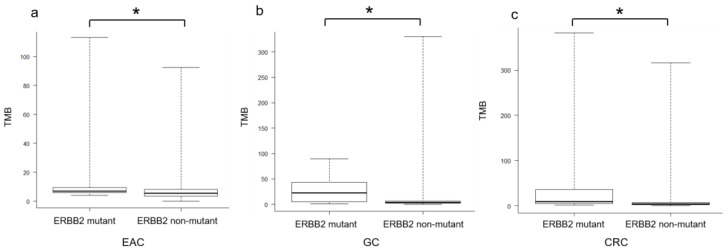
Comparison of tumor mutation burden (TMB) in ERBB2-mutant and ERBB2-non-mutant EAC, GC, and CRC. (**a**) TMB in ERBB2-mutant (*n* = 22) and ERBB2-non-mutant (*n* = 293) EAC. The mean TMB for ERBB2-mutant and ERBB2-non-mutant EAC was 21.0 and 6.5 mut/Mb, respectively (*p* < 0.05). (**b**) TMB in ERBB2-mutant (*n* = 38) and ERBB2-non-mutant (*n* = 549) GC. The mean TMB for ERBB2-mutant and ERBB2-non-mutant GC was 26.9 and 10.0 mut/Mb, respectively (*p* < 0.05). (**c**) TMB in ERBB2-mutant (*n* = 39) and ERBB2-non-mutant (*n* = 1026) CRC. The mean TMB for ERBB2-mutant and ERBB2-non-mutant CRC was 40.0 and 10.5 mut/Mb, respectively (*p* < 0.05). Statistical analysis was performed using the Mann–Whitney U test, and significant differences (*p* < 0.05) are denoted using asterisks (*).

EAC, GC, and CRC presented significantly higher MSI and TMB in the *ERBB2*-mutant group than in the *ERBB2*-wild-type group (*p* < 0.05) (Table 1, Figure 1). In patients with GC and CRC, the proportions of TMB-high tumors were significantly higher in the *ERBB2*-mutant group than in the *ERBB2*-wild-type group (*p* < 0.05), whereas, in patients with EAC, no significant difference was found between the groups (*p* = 0.11). In contrast, regarding the MS status, 17 cases (77.3%) of *ERBB2*-mutant EAC showed MSS; 17 cases (77.3%) were also TMB-low. Fourteen cases (56%) of GC showed MSS, and 17 cases (44.7%) were TMB-low. Twenty-nine cases (74.4%) of CRC showed MSS, and 26 cases (66.7%) were TMB-low.

### 3.2. Classification of ERBB2-Mutant EAC, GC, and CRC and Clinicopathological Features of the Groups

We identified 21 samples of *ERBB2*-mutant EAC among 315 EAC samples (7.0%); 8/315 (2.5%) had coexisting *ERBB2* mutations and amplifications. *ERBB2* mutations were found in 38 of 587 GC cases (6.9%) and 4/587 (0.7%) cases had both *ERBB2* mutations and amplifications. *ERBB2* mutations were found in 39 of 1065 CRC cases (3.7%), and 6/1065 (0.6%) cases had coexisting mutations and amplifications. EAC, GC, and CRC were divided into three groups: group 1 comprised samples with both *ERBB2* mutations and amplifications, group 2 comprised samples with *ERBB2* mutations annotated as putative driver mutations by OncoKB but without amplifications, and group 3 consisted of samples evaluated as non-driver mutations (passenger mutations or unknown significance) by OncoKB and those that were not amplified. The 21 *ERBB2*-mutant EACs, 38 *ERBB2*-mutant GCs, and 39 *ERBB2*-mutant CRCs were divided into groups 1 (*n* = 8, 4, 6, respectively), 2 (*n* = 9, 25, 16, respectively), and 3 (*n* = 5, 9, 17, respectively).

**Figure 2:**
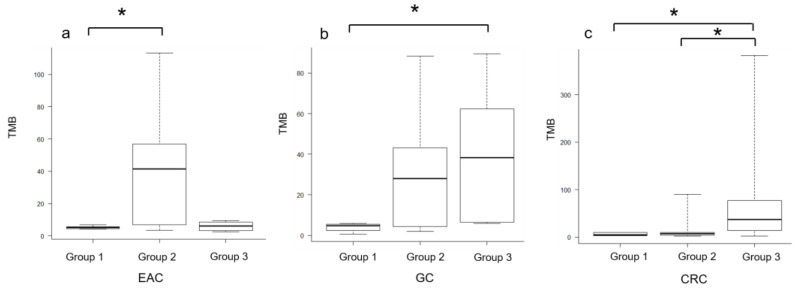
Tumor mutation burden (TMB) among groups 1, 2, and 3 with EAC, GC, and CRC. (**a**) TMB of groups 1, 2, and 3 of ERBB2-mutant EAC. Group 2 showed the highest TMB, although no significant differences were observed among the three groups. (**b**) TMB of groups 1, 2, and 3 of ERBB2-mutant GC. Significant differences were found among the three groups (*p* < 0.05), with groups 2 and 3 showing higher values than group 1. (**c**) TMB of groups 1, 2, and 3 of ERBB2-mutant CRC. Significant differences were found among the three groups (*p* < 0.05), with group 3 showing a higher value than groups 1 and 2. Statistical analysis was performed using the Kruskal–Wallis test, and Steel–Dwass post-test correlation was used to reduce the likelihood of false positives. Significant differences (*p* < 0.05) are denoted using asterisks (*).

For EAC (Table 2), both groups 1 and 3 showed 100% MSS and TMB-low tumors, whereas the proportion of both MSI- and TMB-high tumors was 55.6% in group 2. The MS status and proportions of TMB-low and -high tumors were significantly different among the groups (*p* < 0.05). Although the mean TMB value did not show a significant difference among the groups (*p* > 0.05), group 2 had a higher mean TMB than the other groups, and groups 1 and 2 showed significant differences (*p* < 0.05) (Figure 2a).

For GC (Table 3), group 2 showed a high proportion (73.3%) of grade 3 tumors (*p* < 0.05). The TMB values of groups 2 and 3 were higher than those of group 1 and differed significantly among the three groups (*p* < 0.05); significant differences in TMB were also found between groups 1 and 3 (*p* < 0.05) (Figure 2b). The proportions of TMB-high and TMB-low tumors were also higher in groups 2 and 3 than in group 1 (*p* < 0.05).

For CRC (Table 4), group 3 had the highest number of MSI-type subtypes (54.5%), whereas groups 1 and 2 had the highest number of CIN-type subtypes, with a significant difference among the three groups (*p* < 0.05). Group 1 showed 100% MSS, group 2 showed 87.5% MSS, and group 3 had 47.1% MSI-high tumors, with a significant difference among the groups. Group 3 also had the highest mean TMB value of 78.5 mutations per megabase (mut/Mb); TMB was significantly different among groups 1, 2, and 3 (*p* < 0.05) (Figure 2c). The proportion of TMB-low tumors was 100% in groups 1 and 2, whereas that of TMB-high tumors was 76.5% in group 3, showing a significant difference among the three groups and between groups 1 and 2. 

### 3.3. Genomic Landscape of Somatic Mutations and Comparison of Somatic Mutations among the EAC, GC, and CRC Groups

**Figure 3:**
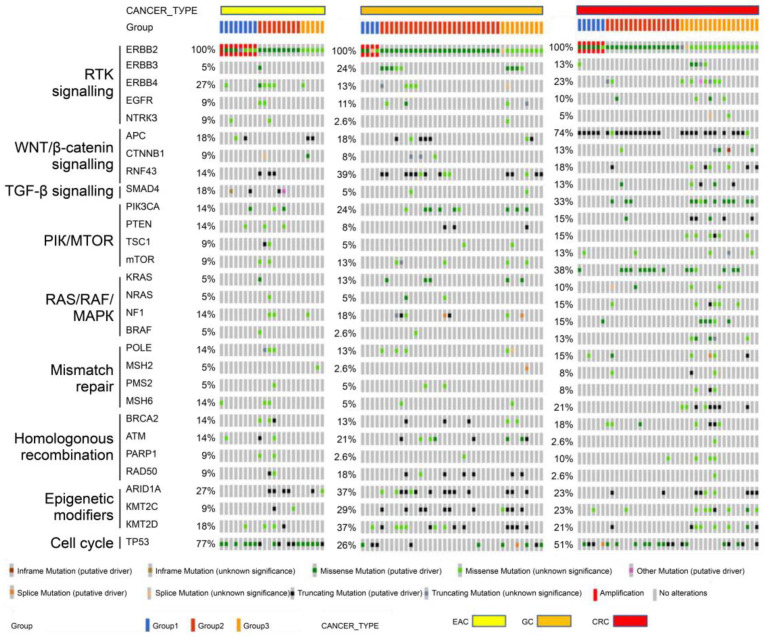
Mutation landscape of *ERBB2*-mutant EAC, GC, and CRC. Co-mutational frequency in *ERBB2*-mutant EAC (*n* = 22), GC (*n* = 38), and CRC (*n* = 39). Oncoprint showing genes across multiple pathways, with altered frequency of mutations, including cancer-related genes. Each column represents one sample. Genes are assorted according to pathways in which they are implicated, with the frequency of the alterations denoted on the left.

Oncoplots summarizing all gene names, as well as the presence/absence, types, and frequency of mutations for each group within the EAC, GC, and CRC groups, are shown in Figure 3. 

The oncoplot showed fewer co-mutations in group 1 than in groups 2 and 3 of EAC, GC, and CRC. In EAC, the mutation rate of MAPK pathway genes downstream to *ERBB2* was 25.0% in group 1, 55.6% in group 2, and 0% in group 3, whereas that of PI3K/MTOR pathway genes was 0% in group 1, 11.1% in group 2, and 20.0% in group 3. In GC, the mutation rate of MAPK pathway genes was 0% in group 1, 40.0% in group 2, and 44.4% in group 3, whereas that of PI3K/MTOR pathway genes was 0% in group 1, 36.0% in group 2 and 22.2% in group 3. In CRC, the mutation rate of MAPK pathway genes was 16.7% in group 1, 25.0% in group 2, and 76.5% in group 3, whereas that of PI3K/MTOR pathway genes was 33.3% in group 1, 62.5% in group 2, and 70.6% in group 3. The proportion of PI3K/MTOR pathway gene mutations to varying degrees was 33.3% in group 1, 62.5% in group 2, and 70.6% in group 3 (Table 5). 

Genetic variations for each tumor group and the results of statistical analysis among the groups are summarized in Table S3.

## 4. Discussion

In this study, we first compared *ERBB2*-mutant and *ERBB2*-non-mutant EAC, GC, and CRC in terms of MS status and TMB using public databases. The *ERBB2*-mutant group had significantly more MSI- and TMB-high tumors than the *ERBB2*-non-mutant group of EAC, GC, and CRC. This result is consistent with that of previous studies [5,13]. As further classification to accurately identify MSI- and TMB-high groups was considered important for the detection of immune checkpoint inhibitor indications in *ERBB2*-mutant tumors, we divided the tumors into three groups according to the presence or absence of *ERBB2* amplifications and annotation content. Although *ERBB2* amplifications and mutations are thought to be mutually exclusive [27,28], in this study, we identified cases of EAC (8/315, 2.5%), GC (4/587, 0.7%), and CRC (6/1065, 0.6%) with simultaneous amplifications and mutations, but at a low frequency. For EAC, the MS status and frequency of TMB-low and -high tumors among the three groups were significantly different. In particular, all patients in groups 1 and 3 had low MSS and a low proportion of TMB-low tumors. For GC, G3 histological grade was more frequently observed in group 2, and it differed significantly among the three groups. Groups 2 and 3 had similar distributions of MS status, mean TMB, and proportions of TMB-low and -high tumors, and, in group 1, all patients had MSS and TMB-low tumors. For CRC, MSI and polymerase epsilon (POLE) subtype were significantly more common in group 3. In addition, the distribution of MS status, average TMB, and proportions of TMB-low and -high tumors showed significant differences among the three groups. For CRC, all patients in group 1 had MSS and TMB-low tumors, whereas, in group 2, one patient (6.3%) presented as MSI-high, and all patients had TMB-low tumors.

We found that the percentages of MSI- and TMB-high tumors were significantly different in each of the three groups of EAC, GC, and CRC. This finding indicates that these three groups are biologically distinct. The division into three groups allowed us to classify the heterogeneous groups to some extent in terms of MSI- and TMB-high. Group 2 of EAC, groups 2 and 3 of GC, and group 3 of CRC had high percentages of MSI- and TMB-high tumors. MSI- and TMB-high are biomarkers of immune checkpoint inhibitor indication [29,30], and this classification scheme allowed us to identify patients with *ERBB2*-mutant tumors who could be candidates for treatment with immune checkpoint inhibitors.

Additionally, we analyzed 28 cancer-associated genetic variants with *ERBB2* mutations. Notably, the *ERBB2* mutations in EAC, GC, and CRC were genetically heterogeneous, with substantial diversity in the co-mutation patterns among carcinomas and within groups. Group 1 had a few comorbid genetic mutations, whereas groups 2 and 3 often presented mutations in other cancer-related genes. Targeting *ERBB2* amplifications and mutations, as in group 1 in this study, a previous study demonstrated the efficacy of trastuzumab [7], with other reports of clinical efficacy [31,32,33]. This may be because, in general, group 1 had fewer concomitant mutations in downstream genes. In contrast, co-mutations were often observed in MAPK and PI3K/MTOR pathway genes downstream of *ERBB2* in groups 2 and 3 of GC and CRC, respectively. These results showed that the mutation status of cancer-related genes in the PI3K/MTOR and MAPK pathways differed among EAC, GC, and CRC, and among the three groups. A gain-of-function mutation in a downstream gene (signal), such as the PI3K/MTOR or MAPK signal targeting only the *ERBB2* mutation, may not stop the entire signal, resulting in reduced sensitivity to the drug. In previous studies, PIK3CA mutations were found in 21.4% of *ERBB2*-mutant tumors [34] and 23.8% of *ERBB2*-mutant CRC [5], which, along with our results, suggests that PIK3CA is a relatively common gene co-mutated with *ERBB2*. In *ERBB2*-amplified tumors, PIK3CA mutations render anti-HER2 therapy less effective [35], although dual inhibition of *ERBB2* and PIK3CA expression has been reported to overcome resistance to therapy [36]. Furthermore, in breast cancer, mutations in MAPK pathway genes have been shown to impart resistance to anti-HER2 therapy but not to treatment with MEK/ERK inhibitors. Thus, PI3K/MTOR and MEK/ERK inhibitors may exert antitumor effects even in *ERBB2*-mutant tumors when downstream mutations are present [37]. Although *ERBB2* mutations are independent biomarkers for chemotherapy, their clinical application is limited because of variability in the therapeutic response to single agents [11]. 

In this study, *ERBB2*-mutant gastrointestinal tumors (EAC, GC, and CRC) were classified into three groups. The MSI and TMB were different in each group, and the co-mutation patterns of cancer-related genes were also different. In particular, the PI3K/MTOR and MAPK pathways, which are downstream of ERBB2, also showed different mutation rates among the groups. The results of this study suggest that, as ERBB2 mutations are biologically heterogeneous, it is important to consider immune checkpoint inhibitors in groups with high MSI and TMB, or dual inhibition that also inhibits downstream mutations in groups with downstream mutations, rather than targeting ERBB2 mutations alone. 

Our study had certain limitations. First, the percentages calculated in this study may not be accurate as we used different datasets, the data for some of the tested characteristics were not available (N/A), and, for some categories, data were not available in several instances. Second, we categorized heterogeneous *ERBB2*-mutant tumor groups into three groups, each of which was found to have distinct biological characteristics. Furthermore, the appropriate treatment for each group was discussed on the basis of biological characteristics and co-mutated genes of the downstream signaling pathways of *ERBB2*. However, as this study was based on bioinformatics analyses, further investigation is needed using wet laboratory studies and actual clinical trials to validate this classification method. Third, the number of cases was low in some datasets; however, multiple datasets were used, which may have overridden this limitation to some extent. Lastly, the current study included the three gastrointestinal tumors (EAC, GC, and CRC) in the same analysis, reporting that they behaved differently. Therefore, EAC, GC, and CRC must be studied individually in the future.

## 5. Conclusions

In this study, we classified the *ERBB2* mutations in EAC, GC, and CRC into three groups each. The pattern of co-mutation of cancer-related genes varied among carcinomas and the groups, with group 1 showing a low frequency of co-mutated genes in all carcinomas and groups 2 and 3 showing varying degrees of mutations in MAPK and PI3K/MTOR pathway genes downstream to *ERBB2*. *ERBB2*-mutant tumors in this study could be classified into three groups from a biological point of view, with different frequencies of co-mutated genes and different percentages of TMB- and MSI-high. In summary, *ERBB2*-mutant tumors proved to be biologically heterogeneous. In the future, treatment methods may be tailored to the biological features of *ERBB2*-mutant tumors, and such classification may contribute to the development of more effective therapies for *ERBB2*-mutant gastrointestinal tumors.

## Figures and Tables

**Table 1:** Comparison of MS and TMB between ERBB2-mutant and ERBB2-wild-type EAC, GC, and CRC.

Cancer Type	Characteristic	Category	*ERBB2*-Mutant	*ERBB2*-Wild-Type	*p*
EAC	MS status (%)	MSS	17 (77.3)	272 (95.1)	<0.05
		MSI-I	0 (0)	3 (1.0)	
		MSI-H	5 (22.7)	11 (3.8)	
		N/A	0	7	
	TMB (mut/Mb)	mean	22.7	7.2	<0.05
		SD	33.4	9.1	
		Median	6.9	5.4	
		Minimum	4.1	0	
		Maximum	113.3	92.5	
		Low (<10)	17 (77.3)	265 (90.4)	0.11
		High (≥10)	5 (22.7)	28 (9.6)	
GC	MS status (%)	MSS	14 (56)	342 (82.8)	<0.05
		MSI-I	2 (8)	11 (2.7)	
		MSI-H	9 (36)	60 (14.5)	
		N/A	13	136	
	TMB (mut/Mb)	mean	26.9	10.0	<0.05
		SD	25.7	23.3	
		Median	22.5	3.5	
		Minimum	0.7	0	
		Maximum	89.6	330.8	
		Low (<10)	17 (44.7)	453 (83.1)	<0.05
		High (≥10)	21 (55.2)	92 (16.9)	
		N/A	0	4	
CRC	MS status (%)	MSS	29 (74.4)	894 (88.7)	<0.05
		MSI-I	1 (2.6)	19 (1.9)	
		MSI-H	9 (23.1)	95 (9.4)	
	TMB (mut/Mb)	mean	40.0	10.5	<0.05
		SD	80.2	23.1	
		Median	9.8	4.6	
		Minimum	2.0	0	
		Maximum	382.6	316.8	
		Low (<16)	26 (66.7)	870 (90.0)	<0.05
		High (≥16)	13 (33.3)	96 (9.9)	

EAC: esophageal adenocarcinoma, GC: gastric cancer, CRC: colorectal cancer, mut: mutation, MS: microsatellite, MSS: MS stability, MSI-I: MS instability—intermediate, MSI-H: MS instability—high, TMB: tumor mutation burden, SD: standard deviation, N/A: not available, Mb: megabase.

**Table 2:** Clinicopathological information of the three groups with *ERBB2*-mutant EAC.

Characteristic	Category	Group 1 (*n* = 8)	Group 2 (*n* = 9)	Group 3 (*n* = 5)	*p*
Age (years)	Mean	65.8	69.0	67.0	0.81
	N/A	2	6	3	
Sex (%)	Male	6 (75.0)	6 (66.7)	4 (80.0)	1
	Female	2 (25.0)	3 (33.3)	1 (20.0)	
Histological grade (%)	G1	1 (12.5)	2 (22.5)	0 (0)	0.28
	G2	5 (62.5)	5 (55.6)	1 (20.0)	
	G3	2 (25.0)	2 (22.2)	4 (80.0)	
Subtype (%)	CIN	6 (100)	1 (33.3)	2 (100)	0.07
	GS	0 (0)	0 (0)	0 (0)	
	MSI	0 (0)	2 (66.7)	0 (0)	
	POLE	0 (0)	0 (0)	0 (0)	
	N/A	2	6	3	
MS status (%)	MSS	8 (100)	4 (44.4)	5 (100)	<0.05
	MSI-I	0 (0)	0 (0)	0 (0)	
	MSI-H	0 (0)	5 (55.6)	0 (0)	
TMB (mut/Mb)	Mean	5.2	43.5	6.0	0.05
	SD	1.0	42.9	3.2	
	Median	5.0	41.5	6.1	
	Minimum	4.1	3.5	2.4	
	Maximum	6.9	113.2	9.5	
	Low (<10)	8 (100)	4 (44.4)	5 (100)	<0.05
	High (<10)	0 (0)	5 (55.6)	0 (0)	

CIN: chromosomal instability, GS: genome stability, POLE: polymerase epsilon, MS: microsatellite, MSS: MS stability, MSI-I: MS instability—intermediate, MSI-H: MS instability—high, TMB: tumor mutation burden, SD: standard deviation, N/A: not available.

**Table 3:** Clinicopathological information on the three groups with *ERBB2*-mutant GC.

Characteristic	Category	Group 1 (*n* = 4)	Group 2 (*n* = 25)	Group 3 (*n* = 9)	*p*
Age (years)	Mean	72.3	68.1	64.3	0.32
	N/A	0	2	2	
Sex (%)	Male	2 (50)	10 (43.5)	6 (66.7)	0.78
	Female	2 (50)	13 (56.5)	3 (37.5)	
	N/A	0	2	1	
Histological grade (%)	G1	0 (0)	0 (0)	0 (0)	<0.05
	G2	3 (100)	4 (26.7)	5 (71.4)	
	G3	0 (0)	11 (73.3)	2 (28.6)	
Subtype (%)	CIN	2 (100)	5 (22.7)	3 (33.3)	0.32
	EBV	0 (0)	1 (4.5)	0 (0)	
	GS	0 (0)	1 (4.5)	1 (11.1)	
	MSI	0 (0)	15 (68.2)	5 (56.6)	
	N/A	2	3	0	
MS status	MSS	3 (100)	7 (46.7)	4 (57.1)	0.47
	MSI-I	0 (0)	1 (6.7)	1 (14.3)	
	MSI-H	0 (0)	7 (46.7)	2 (28.6)	
	N/A	1	10	2	
TMB (mut/Mb)	Mean	4.0	26.9	36.9	<0.05
	SD	2.3	23.5	31.9	
	Median	4.8	28.0	38.3	
	Minimum	0.7	2.0	5.9	
	Maximum	5.9	88.4	89.6	
	Low (<10)	4 (100)	10 (40.0)	3 (33.3)	0.07
	High (≥10)	0 (0)	15 (60.0)	6 (66.7)	

CIN: chromosomal instability, GS: genome-stable, POLE: polymerase epsilon, EBV: Epstein–Barr virus, MS: microsatellite, MSS: MS stability, MSI-I: MS instability-intermediate, MSI-H: MS instability-high, TMB: tumor mutation burden, SD: standard deviation, N/A: Not available.

**Table 4:** Clinicopathological information on the three groups with *ERBB2*-mutant CRC.

Characteristic	Category	Group 1 (*n* = 6)	Group 2 (*n* = 16)	Group 3 (*n* = 17)	*p*
Age (years)	Mean	58.3	65.5	62.7	0.67
Sex (%)	Male	5 (83.3)	9 (56.3)	10 (58.8)	0.56
	Female	1 (16.7)	7 (43.8)	7 (41.2)	
Histological grade	G1	0 (0)	2 (12.5)	1 (5.9)	0.73
	G2	4 (66.7)	9 (56.3)	13 (76.5)	
	G3	2 (33.3)	5 (31.3)	3 (17.6)	
Subtype (%)	CIN	3 (100)	4 (80.0)	2 (18.2)	<0.05
	GS	0 (0)	1 (20.0)	1 (9.1)	
	MSI	0 (0)	0 (0)	6 (54.5)	
	POLE	0 (0)	0 (0)	2 (18.2)	
	N/A	3	11	6	
MS status	MSS	6 (100)	14 (87.5)	9 (52.9)	<0.05
	MSI-I	0 (0)	1 (6.3)	0 (0)	
	MSI-H	0 (0)	1 (6.3)	8 (47.1)	
TMB (mut/Mb)	Mean	5.9	11.9	78.5	<0.05
	SD	3.2	21.0	109.8	
	Median	4.7	7.3	37.0	
	Minimum	2.9	2.0	2.0	
	Maximum	9.8	89.9	382.6	
	Low (<16)	6 (100)	16 (100)	4 (23.5)	<0.05
	High (≥16)	0 (0)	0 (0)	13 (76.5)	<0.05

CIN: chromosomal instability, GS: genome stable, POLE: polymerase epsilon, MS: microsatellite, MSS: MS stability, MSI-I: MS instability—intermediate, MSI-H: MS instability—high, TMB: tumor mutation burden, SD: standard deviation.

**Table 5:** PI3K/MTOR and MAPK pathway mutation rates in the three groups with EAC, GC, and CRC.

Cancer Type	Group (Number)	PI3K/MTOR (%)	MAPK (%)
EAC			
	Group 1 (*n* = 8)	2 (25.0)	0 (0)
	Group 2 (*n* = 9)	5 (55.6)	1 (11.1)
	Group 3 (*n* = 5)	0 (0)	1 (20.0)
GC			
	Group 1 (*n* = 4)	0 (0)	0 (0)
	Group 2 (*n* = 25)	10 (40.0)	9 (36.0)
	Group 3 (*n* = 9)	4 (44.4)	2 (22.2)
CRC			
	Group 1 (*n* = 6)	1 (16.7)	2 (33.3)
	Group 2 (*n* = 16)	4 (25.0)	10 (62.5)
	Group 3 (*n* = 17)	13 (76.5)	12 (70.6)

EAC: esophageal adenocarcinoma, GC: gastric cancer, CRC: colorectal cancer, PI3K: phosphoinositide 3-kinase, MTOR: mammalian target of rapamycin, MAPK: mitogen-activated protein kinase.

## Data Availability

The datasets generated and/or analyzed in this study are available from the corresponding author on reasonable request.

## Supplementary Materials

The following supporting information can be downloaded at https://www.mdpi.com/article/10.3390/cimb45090468/s1: Table S1. Mutation types and annotation results for *ERBB2* mutations in EAC, GC, and CRC; Table S2. Summary of *ERBB2* mutation sites in the three groups of EAC, GC, and CRC; Table S3. Mutation frequencies of cancer-related genes in the groups with EAC, GC, and CRC and results of statistical analysis.
